# Association between A Family History of Colorectal Cancer and the Risk of Colorectal Cancer: A Nationwide Population-Based Study

**DOI:** 10.3390/jpm12101566

**Published:** 2022-09-23

**Authors:** Yoon Suk Jung, Huiyeon Song, Mai Thi Xuan Tran, Boyoung Park, Chang Mo Moon

**Affiliations:** 1 Division of Gastroenterology, Department of Internal Medicine, Kangbuk Samsung Hospital, Sungkyunkwan University School of Medicine, Seoul 03181, Korea; 2 Department of Epidemiology and Biostatistics, Graduate School of Public Health, Hanyang University, Seoul 04763, Korea; 3 Department of Preventive Medicine, Hanyang University College of Medicine, Seoul 04763, Korea; 4 Department of Internal Medicine, College of Medicine, Ewha Womans University, Seoul 07985, Korea; 5 Inflammation-Cancer Microenvironment Research Center, College of Medicine, Ewha Womans University, Seoul 07804, Korea

**Keywords:** family history, colorectal cancer, age

## Abstract

Large-scale Asian studies on this topic are lacking. We evaluated the CRC risk associated with family history in the Korean population. We analyzed the data of participants aged ≥40 years who underwent national cancer screening between 2013 and 2014. During a mean follow-up of 4.7 ± 0.8 years, 0.43% of the 292,467 participants with family history and 0.28% of the 1,169,868 participants without family history developed CRC. Participants with a family history in any FDR, parents only, and siblings only had a higher risk of CRC than those without family history; adjusted hazard ratios (HRs) were 1.53, 1.46, and 1.61, respectively. Participants with a family history comprising both parents and siblings had an even higher risk of CRC than those without a family history (HR, 2.34). The HRs for CRC in the 40–49, 50–59, 60–69, 70–79, and ≥80 age groups with family history were 1.72, 1.74, 1.50, 1.30, and 0.78, respectively (*p* < 0.001). A family history of CRC in any FDR and both parents and siblings was associated with an approximately 1.5- and 2.3-fold increased risk of CRC. The effect of family history was relatively greater in the younger than the older age group.

## 1. Introduction

Colorectal cancer (CRC) is the third most commonly diagnosed cancer and the second leading cause of cancer death globally [1]. In 2020, an estimated 1,880,725 new CRC cases and 915,880 CRC deaths occurred worldwide [1]. A family history of CRC is a well-known risk factor for CRC. Based on recent meta-analyses, a family history of CRC in a first-degree relative (FDR) is associated with an approximately 1.8- to 1.9-fold higher risk of developing CRC [2,3]. Accordingly, individuals with a family history of CRC have been regarded as a high-risk group for CRC, with the guidelines recommending more aggressive CRC screening strategies for these individuals [4,5]. For instance, the American College of Gastroenterology CRC screening guideline recommends initiating CRC screening with fecal immunochemical testing (FIT) or colonoscopy at age 50 for average-risk individuals, and initiating CRC screening with a colonoscopy at age 40, or 10 years before the youngest affected relative, whichever is earlier, for individuals with a family history of CRC or advanced polyp in one FDR at younger than 60 years, or CRC or advanced polyp in ≥ 2 FDRs diagnosed at any age [4]. However, these recommendations for individuals with a family history of CRC are based on low-quality evidence. In addition, these recommendations are based on studies from Western countries, as most studies on the relationship between family history and CRC risk were conducted in Western countries. Accordingly, limited data have been reported on this topic in Asian countries. In a recent meta-analysis, the number of patients in Asian studies was one-tenth that in non-Asian studies, clearly demonstrating the relative lack of studies with Asian cohorts [3].

Several large cohort studies from Western countries reported that the higher the number of affected FDRs with CRC, the greater the risk for CRC. Further, the risk of CRC associated with a family history is higher in younger individuals than in older individuals [6,7,8]. However, it is not clear whether such results are similar for Asians. Considering the current trend of increasing CRC incidence in Asian countries [9], highly valid large cohort studies from Asian countries are increasingly necessary. 

We conducted this population-based study using large-scale data from a national health insurance database to clarify the association between family history of CRC and the risk of CRC, and to evaluate the risk of CRC according to the characteristics of the affected FDR in the Korean population. 

## 2. Materials and Methods

### 2.1. Data Source of the Study

Customized data from the National Health Information Database (NHID) embedded in the National Health Insurance Service (NHIS) in Korea were used in this study. All Korean citizens were covered by the NHIS for medical service use. Based on the fee-for-service system and payment contribution according to income and property, the NHIS includes sociodemographic information, medical utilization from clinics and pharmacies, survival status, and results of national health screenings [10]. Biennial upper endoscopy or upper gastrointestinal series for people ≥ 40 years and annual fecal occult blood tests for people ≥ 50 years are provided as part of national gastric and CRC screening programs [11]. This study was approved by the Institutional Review Board of Ewha Womans University Mokdong Hospital (approval no. 2020-08-030). Based on the agreement to transfer their data to the NHID at screening, the requirement for informed consent was waived, and an anonymized database was constructed.

### 2.2. Study Population

**Figure 1:**
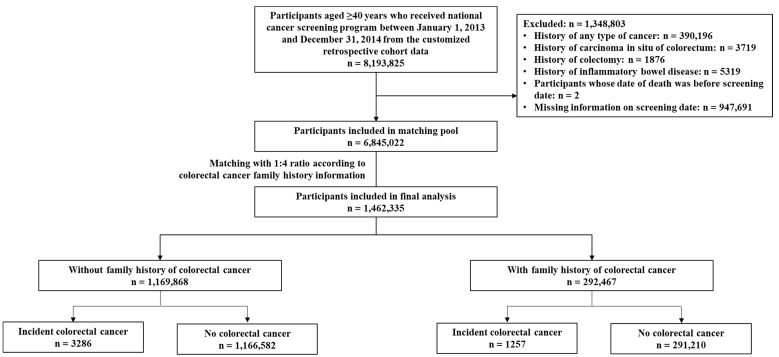
Flowchart of the study population.

A total of 8,193,825 men and women aged ≥ 40 years who underwent a national cancer screening program between 1 January 2013 and 31 December 2014 were included in this study. To identify newly diagnosed cancer cases, we excluded individuals who had a history of any type of cancer (N = 390,196) or carcinoma in situ in the colon, rectosigmoid junction, or rectum (N = 3719) before the screening, based on medical utilizations or self-reported questionnaires, and those who underwent colectomy before the screening (N = 1876). As a high-risk group for CRC, participants with a history of inflammatory bowel disease before screening were excluded (N = 5319). In addition, participants whose recorded date of death was before the screening date (N = 2) and those with missing information on the screening date (N = 947,691) were excluded, leaving 6,845,022 participants (Figure 1). Patients were followed until CRC diagnosis, death, or 31 December 2018, whichever came first.

### 2.3. Assessment of Family History of CRC in FDRs and Outcome

Information on the history of cancer and cancer type among FDRs (parents, siblings, and children) was obtained from the NHIS-NHID using a standardized questionnaire during cancer screening. Participants with a family history of CRC in any FDR were identified based on the questionnaire information.

The incidence of CRC or carcinoma in situ in the colon, rectosigmoid junction, or rectum was defined based on the corresponding International Classification of Diseases 10th revision (ICD-10) code (C18, C19, C20, D01.0, D01.2, and D01.3) and the catastrophic illness code for cancer. The catastrophic illness code is associated with a waiver of co-payment for diagnostic procedures and treatments for cancer in Korea. The accuracy of the ICD-10 code for CRC with a catastrophic illness code compared to the Korean Cancer Registry was up to 92.3% [12].

### 2.4. Covariates

The following factors were considered as covariates: age at screening, sex, obesity status (defined as a body mass index (BMI) ≥25 kg/m^2^), smoking habits (never smoker, former smoker, and current smoker), alcohol consumption (one time or less of alcohol drinking and two times or more of alcohol drinking per week), physical activity (no light or vigorous walk per week and one or more light or vigorous walks per week), aspirin use (prescription of low-dose aspirin for 104 days or more during the follow-up, similar to a previous study [13]), and presence of comorbidities (including hypertension, diabetes mellitus, dyslipidemia, ischemic heart disease, and stroke). Information on smoking habits, alcohol consumption, physical activity, and the presence of comorbidities was obtained using a self-administered questionnaire from the NHIS-NHID. BMI was calculated based on measured height and weight during the health examination, and aspirin use was identified from the prescription records in medical utilization databases.

### 2.5. Matching between Individuals with and without a Family History of CRC

To increase comparability, frequency matching according to the 5-year age group and sex with a ratio of 1:4 between individuals with and without a family history of CRC was performed. The final study population comprised 292,467 participants with a family history of CRC and 1,169,868 participants without a family history of any type of cancer (Figure 1).

### 2.6. Statistical Analysis

Descriptive statistics of the baseline characteristics were compared between those with and without a family history of CRC using the Student’s t-test or chi-squared test. Continuous variables are presented as mean ± standard deviation, while categorical variables are presented as number (percent). CRC risk is presented in terms of incidence, crude hazard ratios (HRs), adjusted HRs, and 95% confidence intervals (CIs). The incidence rate was calculated as cases per 10,000 person-years, and adjusted HRs were estimated using the Cox proportional hazards regression model adjusted for the above-mentioned covariates. Tests for the assumption of proportional hazards were performed using Kaplan–Meier curves, and parallel lines of the log–log survival functions were identified. The cumulative incidence of CRC according to a family history of CRC was calculated, and the Gray test was applied to compare the cumulative function. Statistical analyses were performed using the SAS statistical software (version 9.4; SAS Institute, Cary, NC, USA).

## 3. Results

### 3.1. Baseline Characteristics of the Study Population

Table 1 shows the baseline characteristics of the study population. The mean age of the study participants at screening was 53.8 ± 9.7 years, and 42.7% of the participants were men. Participants without a family history of CRC had higher rates of obesity, never smokers, and aspirin use than those with a family history of CRC. Participants with a family history of CRC also had higher rates of alcohol consumption and physical activity than those without a family history of CRC.

### 3.2. Association between Family History of CRC and the Risk of CRC 

The mean follow-up period was 4.7 ± 0.8 years (median 4.7 years, interquartile range 4.2–5.2 years). The follow-up period was similar between participants with and without a family history of CRC (4.7 ± 0.9 vs. 4.7 ± 0.8 years). During the follow-up period, among the 1,169,868 participants without a family history of CRC, 3286 (0.28%) developed CRC and among the 292,467 participants with a family history of CRC, 1257 (0.43%) developed CRC. The mean age at CRC diagnosis in the people with family history of CRC was 61.7 ± 10.2 years and that in those without family history of CRC was 63.3 ± 9.7 years. The factors associated with CRC risk are shown in Table 2.

After adjusting for all variables, a family history of CRC was identified as a significant risk factor for CRC (adjusted HR 1.53, 95% CI 1.43–1.63). Age at screening, male sex, current smoking, and alcohol consumption were independent risk factors for CRC, whereas aspirin use was identified as a significant protective factor against CRC. 

### 3.3. Risk of CRC According to Affected Family Members

Table 3 shows the risk of CRC according to the specific FDR type. Participants with a family history of CRC in parents only and siblings only had a higher risk of CRC than those without a family history of CRC; the corresponding adjusted HRs (95% CIs) were 1.46 (1.34–1.59) and 1.61 (1.47–1.76), respectively. Participants with a family history of CRC in both parents and siblings had an even higher risk of CRC than those without a family history of CRC, with an adjusted HR of 2.34 (95% CI 1.55–3.52). The risk of CRC in participants with a family history of CRC in children only tended to be higher than that in participants without a family history of CRC; however, no statistically significant difference was found (adjusted HR 1.32, 95% CI 0.93–1.87). 

**Figure 2:**
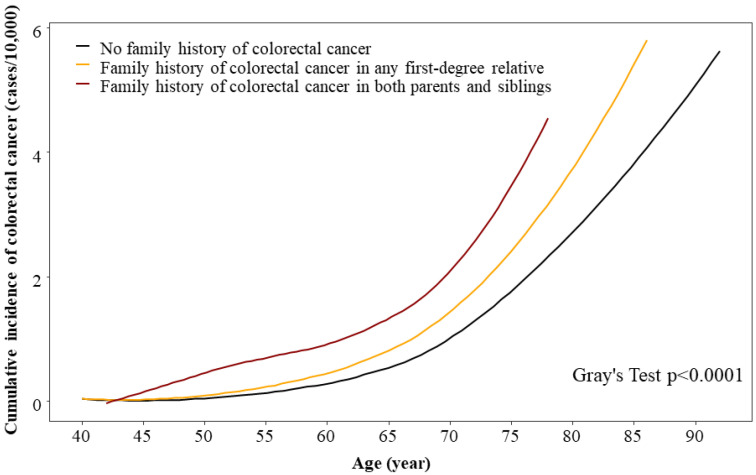
Cumulative incidence of colorectal cancer (CRC) according to a family history of CRC.

The cumulative incidence of CRC according to age and the presence and type of a family history of CRC is shown in Figure 2. The cumulative incidence of CRC was higher in participants with a family history of CRC, particularly when both parents and siblings were affected by CRC. 

### 3.4. Association between a Family History of CRC and the Risk of CRC According to the Age of Participants at Screening

We assessed the age-specific risk of CRC associated with a family history of CRC (Table 4). Among women, the risk of CRC associated with a family history of CRC was highest for the 40–49 years age group. The risk was found to decrease progressively for older women. In particular, the adjusted HRs (95% CIs) for CRC in women aged 40–49, 50–59, 60–69, 70–79, and ≥80 years with a family history of CRC versus all women without a family history of CRC were 1.92 (1.55–2.38), 1.66 (1.42–1.94), 1.57 (1.34–1.83), 1.49 (1.23–1.81), and 1.06 (0.67–1.69), respectively. This trend in family history-associated CRC risk according to age among women was statistically significant (*p* < 0.001). A similar trend was observed in men. In men, the adjusted HRs for CRC related to a family history of CRC also significantly decreased with increasing age (*p* < 0.001), and a family history of CRC was not associated with a significant elevation in the risk of CRC among men aged ≥70 years. For all participants, the adjusted HRs (95% CI) for CRC in the 40–49, 50–59, 60–69, 70–79, and ≥80 age groups were 1.72 (1.48–2.01), 1.74 (1.57–1.93), 1.50 (1.35–1.67), 1.30 (1.13–1.50), and 0.78 (0.54–1.13), respectively, compared to the total participants without a family history of CRC (*p* < 0.001).

## 4. Discussion

In this large population-based study, we found that a family history of CRC in an FDR was associated with an increased CRC risk. The risk of CRC was even higher in individuals with a family history of CRC in both parents and siblings, suggesting familial aggregation of CRC. Notably, the effect of a family history of CRC on the risk of CRC was relatively greater in the younger age groups than in the older age groups. The risk of CRC related to family history gradually decreased in the older age group and did not increase significantly in men >70 years and women >80 years of age.

Many studies have reported that a family history of CRC is closely associated with an increased risk of CRC. However, large-scale studies on this topic have mainly been conducted in Western countries. As Asians have a lower prevalence and incidence of CRC and different genetic and environmental backgrounds [14] than Westerners, the effect of family history on CRC risk may differ between Asians and Westerners. Nevertheless, no large-scale cohort studies with high validity have been conducted in Asian countries. To date, Asian studies on this topic have analyzed hundreds or, at most, thousands of people with a family history of CRC. Accordingly, the reported family history-related CRC risk extensively varies across studies [3]. A recent meta-analysis including 18 Asian studies revealed that a family history of CRC in an FDR was associated with an increased risk of CRC, with a relative risk (RR) of 1.83 (95% CI 1.54–2.16) [3]. In that study, a meta-analysis including 28 non-Asian studies also reported a similar RR of 1.88 (95% CI 1.63–2.17) [3]. However, the number of patients in the Asian studies included in this meta-analysis was markedly smaller than that in the non-Asian studies. Despite pooling the patients included in the 18 Asian studies, only 10,665 individuals with a family history of CRC could be analyzed, and most of the included Asian studies were case-control studies [3]. In contrast, our study evaluated 292,467 individuals with a family history of CRC, which is nearly 30 times more than the total in the prior study. Accordingly, the results of our large-scale analysis may provide more reliable information on the effects of family history of CRC. In our study, a family history of CRC was associated with a 1.53-fold increased risk of CRC (95% CI 1.43–1.63). The risk of CRC associated with a family history of CRC in our study was slightly lower than that reported in previous studies or the above-mentioned meta-analysis [3]. This finding may be because of the short follow-up observation period for CRC occurrence in our study. However, our results suggest that the impact of family history on CRC in Koreans may be slightly lower than that in other countries.

In the present study, the increased risk of CRC was found to vary according to the type of family member. The increased risk of CRC was greater in individuals with a family history of CRC in both parents and siblings (2.3-fold) than in those with a family history of CRC in parents only (1.5-fold) and siblings only (1.6-fold). Although the exact number of family members with CRC could not be confirmed from the questionnaire items, individuals with a family history of CRC in both parents and siblings had at least two affected FDRs, whereas those with a family history of CRC in parents only or siblings only were more likely to have had one affected FDR. Our results may support the findings of previous studies that reported that the greater the number of affected FDRs with CRC, the higher the risk of developing CRC [6,7,15,16]. Our findings support current guidelines that recommend more intensive CRC screening, such as earlier initiation of screening, screening with colonoscopy rather than FIT, and shorter screening intervals (colonoscopy every 5 years instead of every 10 years), for individuals with a family history of CRC in FDRs. In particular, these intensive CRC screening strategies may have to be applied more strictly to individuals with a family history of CRC in both parents and siblings. However, since the number of these individuals in our study was only 23, further studies involving more individuals with multiple FDRs affected by CRC are needed.

In the present study, the association between a family history of CRC and the risk of CRC was stronger in younger age groups than in older age groups. Compared with the total participants without a family history, the adjusted HRs for CRC gradually decreased progressively in older age groups with a family history of CRC, approaching 1 in men ≥70 years and women ≥80 years of age. A family history of CRC was not associated with a significant increase in CRC risk among older age groups. More specifically, the adjusted HRs (95% CIs) for CRC in men aged 70-79 years and ≥80 years with a family history of CRC vs. total men without a family history were 1.14 (0.94–1.40) and 0.54 (0.29–1.01), respectively, and the adjusted HR (95% CIs) for CRC in women aged ≥80 years with a family history of CRC vs. total women without a family history was 1.06 (0.67–1.69). Our results suggest that more intensive colonoscopy screening should be offered to individuals with a positive family history of CRC, particularly at younger ages. Similar to our results, some studies and meta-analyses revealed that the risk of CRC decreases as the age of the at-risk individual increases. A previous US study reported that the effect of a family history of CRC on the risk of CRC was greatest for individuals <45 years, whereas a family history of CRC was not related to a significant increase in risk among individuals ≥60 years [8]. Another US multicenter prospective study involving 144,768 participants revealed that after the age of 55 years, those with one FDR with CRC had only a modest increased risk of developing CRC compared to those without a family history of CRC (HR 1.30; 95% CI 1.10–1.50) [7]. Consistently, a recent meta-analysis of 17 studies demonstrated that family history-associated CRC risk was higher in individuals younger than 50 years than those older than 50 years (RR 2.81; 95% CI 1.94–4.07 for <50 years vs. RR 1.47; 95% CI 1.28–1.69 for ≥50 years; *p* = 0.001) [2]. In addition, a cost-effectiveness analysis study estimated that the risk of developing CRC in an individual with one affected FDR decreased with increasing age from 5.5-fold for ages 30–44 years to no difference (1.1-fold) at ≥70 years [17]. This study suggested that for individuals with one affected FDR, screening every 3 years, beginning at age 40, is most cost-effective; if no colorectal adenomas are found, the CRC screening interval can gradually be extended to 5 and 7 years at the ages of 45 and 55 years, respectively [17]. Unfortunately, we did not perform a cost-effectiveness analysis in this study. Cost-effectiveness studies are needed to establish optimized CRC screening strategies for Koreans with a family history of CRC. Notably, our large-scale data could serve as a foundation for future studies.

To our knowledge, this is the first population-based study to assess the impact of a family history of CRC on the risk of CRC depending on the affected FDRs in Korea. This study is also the largest study on this topic in Asia. However, our study had some limitations. First, as information on the family history of CRC was self-reported, underreporting and recall bias may have been present. Second, the risk of CRC according to the accurate number of affected FDRs and their age at CRC diagnosis was not evaluated. Despite the fact that age at screening was matched between people with and without family history of CRC, the age at CRC diagnosis was lower in people with family history of CRC compared with those without family history (mean age 63.3 vs. 61.7 years). Thus, we considered that the associations in this study were underestimated, showing conservative results. In addition, the association between a family history of CRC in second-degree relatives and the risk of CRC was not assessed. Third, the effect of a family history of advanced colorectal adenoma was not investigated in this study, although the guidelines consider family history of advanced adenoma [4,5]. Additionally, we cannot evaluate cancer stage due to lack of data. Fourth, we cannot exclude hereditary syndromes of CRC, such as Lynch syndrome or familial adenomatous polyposis, which cannot be clearly defined by the ICD-10 code. Lastly, the follow-up observation period for CRC occurrence was short, and individuals younger than 40 years were not analyzed. 

## 5. Conclusions

In conclusion, individuals with a family history of CRC had an approximately 1.5-fold increased risk of CRC compared with those without a family history of CRC. Moreover, a family history of CRC in both parents and siblings was associated with a 2.3-fold higher risk of CRC development. The association between family history of CRC and the risk of CRC was relatively stronger in the younger age groups than in the older age groups. As the risk of CRC associated with a family history of CRC depends on the age of the person to be screened and the number of affected FDRs, guidelines may be improved through a more detailed grouping of family history. 

## Figures and Tables

**Table 1:** Baseline characteristics of study participants according to family history of CRC.

Characteristics	Individuals with a Family History of CRC n = 292,467	Individuals without a Family History of CRC n = 1,169,868	*p*-Value
**Age at screening (years)**	53.8 ± 9.7	53.8 ± 9.7	0.008
**Age group at screening**			
40–49	105,787 (36.2)	423148 (36.2)	
50–59	105,758 (36.2)	423,032 (36.2)	
60–69	57,849 (19.8)	231,396 (19.8)	
70–79	20,678 (7.1)	82,712 (7.1)	
≥80	2395 (0.8)	9580 (0.8)	
**Sex**			
Female	167,532 (57.3)	670,128 (57.3)	
Male	124,935 (42.7)	499,740 (42.7)	
**Obesity**			
<25 kg/m^2^	192,421 (65.8)	758,627 (64.8)	<0.0001
≥25 kg/m^2^	100,013 (34.2)	411,052 (35.1)	
Missing	33 (0.0)	189 (0.0)	
**Smoking habit**			
Never	190,587 (65.2)	784,147 (67.0)	<0.0001
Former	50,157 (17.2)	170,734 (14.6)	
Current	51,627 (17.7)	214,448 (18.3)	
Missing	96 (0.0)	539 (0.0)	
**Alcohol consumption**			
≤1 time per week	219,385 (75.0)	887,777 (75.9)	<0.0001
≥2 times per week	72,793 (24.9)	281,038 (24.0)	
Missing	289 (0.1)	1053 (0.1)	
**Physical activity**			
No	58,960 (20.2)	277,906 (23.8)	<0.0001
≥1 time per week	233,112 (79.7)	890,574 (76.1)	
Missing	395 (0.1)	1388 (0.1)	
**Aspirin use**			
No	260,873 (89.2)	1,037,756 (88.7)	<0.0001
Yes	31,594 (10.8)	132,112 (11.3)	
**Comorbidities**			
Hypertension	61,932 (21.2)	259,298 (22.2)	<0.0001
Diabetes mellitus	22,447 (7.7)	96,013 (8.2)	<0.0001
Dyslipidemia	17,348 (5.9)	58,869 (5.0)	<0.0001
Ischemic heart disease	7349 (2.5)	27,914 (2.4)	<0.0001
Stroke	2819 (1.0)	11,582 (1.0)	0.200

CRC, colorectal cancer. Values are presented as mean ± standard deviation or number (%).

**Table 2:** Factors associated with risk for CRC.

Variables	N	Person-Years	Incidence (95% CI) ^a^	Crude HR (95% CI)	Adjusted HR (95% CI) ^b^
Family history of CRC					
No	3286	5,485,865	6.0 (5.8–6.2)	1 (Reference)	1 (Reference)
Yes	1257	1,383,475	9.1 (8.6–9.6)	1.52 (1.43–1.63)	1.53 (1.43–1.63)
**Age at screening, per year**				1.07 (1.06–1.07)	1.08 (1.07–1.08)
Sex					
Female	2120	3,957,881	5.4 (5.1–5.6)	1 (Reference)	1 (Reference)
Male	2423	2,911,460	8.3 (8.0–8.7)	1.55 (1.46–1.64)	1.49 (1.38–1.62)
Obesity					
<25 kg/m^2^	2838	4,469,641	6.3 (6.1–6.6)	1 (Reference)	1 (Reference)
≥25 kg/m^2^	1705	2,398,755	7.1 (6.8–7.4)	1.12 (1.05–1.19)	1.06 (0.99–1.12)
Smoking habit					
Never	2747	4,596,822	6.0 (5.8–6.2)	1 (Reference)	1 (Reference)
Former	864	1,027,020	8.4 (7.9–9.0)	1.40 (1.30–1.51)	1.02 (0.92–1.12)
Current	930	1,242,386	7.5 (7.0–8.0)	1.25 (1.16–1.35)	1.22 (1.11–1.34)
Alcohol consumption					
≤1 time/week	3260	5,208,261	6.3 (6.0–6.5)	1 (Reference)	1 (Reference)
≥2 times/week	1283	1,654,870	7.8 (7.3–8.2)	1.24 (1.16–1.32)	1.23 (1.14–1.32)
Physical activity					
No	1153	1,587,093	7.3 (6.8–7.7)	1 (Reference)	1 (Reference)
≥1 time per week	3386	5,273,630	6.4 (6.2–6.6)	0.88 (0.83–0.94)	0.94 (0.88–1.01)
Aspirin use					
No	3736	6,106,088	6.1 (5.9–6.3)	1 (Reference)	1 (Reference)
Yes	807	763,253	10.6 (9.8–11.3)	1.73 (1.60–1.86)	0.90 (0.83–0.98)
Comorbidities ^c^					
No	2615	4,893,215	5.3 (5.1–5.5)	1 (Reference)	1 (Reference)
Yes	1928	1,976,126	9.8 (9.3–10.2)	1.82 (1.72–1.93)	1.04 (0.98–1.12)

CRC, colorectal cancer; HR, hazard ratio; CI, confidence interval. ^a^ Incidence rate was calculated as cases per 10,000 person-years. ^b^ Adjusted for age of the participants at screening, sex, obesity, smoking habits, alcohol consumption, physical activity, aspirin use, and presence of comorbidities, including hypertension, diabetes mellitus, dyslipidemia, ischemic heart disease, and stroke. Comorbidities were defined as at least one of the following: hypertension, diabetes mellitus, dyslipidemia, ischemic heart disease, and stroke.

**Table 3:** Risk of CRC according to family members affected by CRC.

Variables	N	Person-Years	Incidence (95% CI) ^a^	Crude HR (95% CI)	Adjusted HR (95% CI) ^b^
No family history of CRC	3286	5,485,865	6.0 (5.8–6.2)	1 (Reference)	1 (Reference)
Family history of CRC in any FDR	1257	1,383,475	9.1 (8.6–9.6)	1.52 (1.43–1.63)	1.53 (1.43–1.63)
Specific FDR					
Parents only	631	912,026	6.9 (6.4–7.5)	1.16 (1.06–1.26)	1.46 (1.34–1.59)
Siblings only	571	441,303	12.9 (11.9–14.0)	2.17 (1.99–2.37)	1.61 (1.47–1.76)
Children only	32	14,134	22.6 (14.8–30.5)	3.78 (2.67–5.36)	1.32 (0.93–1.87)
Parents and siblings	23	15,232	15.1 (8.9–21.3)	2.53 (1.68–3.81)	2.34 (1.55–3.52)
Parents and children	0	271	NA	NA	NA
Siblings and children	0	213	NA	NA	NA
Parents, siblings, and children	0	0	NA	NA	NA

CRC, colorectal cancer; CI, confidence interval; HR, hazard ratio; FDR, first-degree relative; NA, not applicable owing to the small sample size. ^a^ Incidence rate was calculated as cases per 10,000 persons. ^b^ Adjusted for age of the participants at screening, sex, obesity, smoking habits, alcohol consumption, physical activity, aspirin use, and presence of comorbidities, including hypertension, diabetes mellitus, dyslipidemia, ischemic heart disease, and stroke.

**Table 4:** Age-specific relative risk of CRC among individuals with a family history of CRC.

Characteristics	N	Person-Years	Incidence (95% CI) ^a^	Age-Adjusted HR (95% CI)	Adjusted HR (95% CI) ^b^
Women					
No family history	1512	3,161,552	4.8 (4.5–5.0)	1 (reference)	1 (reference)
Family history of CRC					
40–49 years	104	272,762	3.8 (3.1–4.5)	1.92 (1.55–2.37)	1.92 (1.55–2.38)
50–59 years	184	290,747	6.3 (5.4–7.2)	1.66 (1.42–1.94)	1.66 (1.42–1.94)
60–69 years	181	163,886	11.0 (9.4–12.7)	1.56 (1.34–1.83)	1.57 (1.34–1.83)
70–79 years	120	61,463	19.5 (16.0–23.0)	1.49 (1.23–1.81)	1.49 (1.23–1.81)
≥80 years	19	7470	25.4 (14.0–36.9)	1.07 (0.67–1.70)	1.06 (0.67–1.69)
*P*-trend					<0.001
Men					
No family history	1774	2,324,313	7.6 (7.3–8.0)	1 (reference)	1 (reference)
Family history of CRC					
40–49 years	97	229,587	4.2 (3.4–5.1)	1.56 (1.26–1.94)	1.58 (1.27–1.96)
50–59 years	231	210,101	11.0 (9.6–12.4)	1.81 (1.57–2.08)	1.81 (1.57–2.08)
60–69 years	201	108,986	18.4 (15.9–21.0)	1.43 (1.23–1.65)	1.44 (1.24–1.66)
70–79 years	110	35,251	31.2 (25.4–37.0)	1.14 (0.93–1.39)	1.14 (0.94–1.40)
≥80 years	10	3221	31.0 (11.8–50.3)	0.53 (0.28–1.00)	0.54 (0.29–1.01)
*P*-trend					<0.001
Total					
No family history	3286	5,485,865	6.0 (5.8–6.2)	1 (reference)	1 (reference)
Family history of CRC					
40–49 years	201	502,349	4.0 (3.4–4.6)	1.72 (1.48–2.00)	1.72 (1.48–2.01)
50–59 years	415	500,848	8.3 (7.5–9.1)	1.74 (1.57–1.93)	1.74 (1.57–1.93)
60–69 years	382	272,873	14.0 (12.6–15.4)	1.50 (1.35–1.67)	1.50 (1.35–1.67)
70–79 years	230	96,714	23.8 (20.7–26.9)	1.30 (1.13–1.49)	1.30 (1.13–1.50)
≥80 years	29	10,691	27.1 (17.3–37.0)	0.77 (0.53–1.11)	0.78 (0.54–1.13)
*p*-trend					<0.001

CRC, colorectal cancer; CI, confidence interval; HR, hazard ratio. ^a^ Incidence rate was calculated as cases per 10,000 persons. ^b^ Adjusted for age at screening, sex, obesity, smoking habits, alcohol consumption, physical activity, aspirin use, and presence of comorbidities, including hypertension, diabetes mellitus, dyslipidemia, ischemic heart disease, and stroke.

## Data Availability

The data presented in this study are available on request from the corresponding author.
